# Uncommon anomalies of the left pulmonary artery during VATS lobectomy for lung cancer

**DOI:** 10.1002/ccr3.2811

**Published:** 2020-03-23

**Authors:** Dario Amore, Umberto Caterino, Dino Casazza, Pasquale Imitazione, Alessandro Saglia, Maria Rosaria Valentino, Alessandro Izzo, Carlo Curcio

**Affiliations:** ^1^ Department of Thoracic Surgery Monaldi Hospital Naples Italy; ^2^ Department of Respiratory Diseases University of Naples Federico II Monaldi Hospital Naples Italy; ^3^ Department of Respiratory Diseases San Giuseppe Moscati Hospital Avellino Italy

**Keywords:** lung cancer, pulmonary artery, VATS lobectomy

## Abstract

The key to avoid intraoperative complications due to failure in the preoperative detection of pulmonary vascular anomalies is to perform a careful hilar dissection.

## Graphical abstract

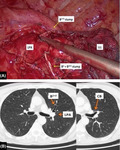

## 1 INTRODUCTION

We herein report two different types of anomalies concerning the left pulmonary artery and recognized in patients undergoing video‐assisted thoracoscopic surgery lobectomy for lung cancer treatment. A detailed knowledge of these anomalies is essential in order to prevent inadvertent division of bronchovascular structures during major anatomic lung resections.

Thoracic surgeons should pay attention to variations of the pulmonary arteries because if these abnormalities are not considered, severe intraoperative complications such as bleeding or greater extension of lung resection may occur.^1^ Although in recent decades several data have demonstrated the advantages of video‐assisted thoracoscopic surgery (VATS) lobectomy, compared with the open approach, minimally invasive procedures for major lung resections sometimes require conversion to thoracotomy and vascular anomalies have been defined as possible risk factors that may be related to conversion in open surgery.^2^, ^3^, ^4^

## 2 CASE REPORT

### 2.1 Case 1

**Figure 1:**
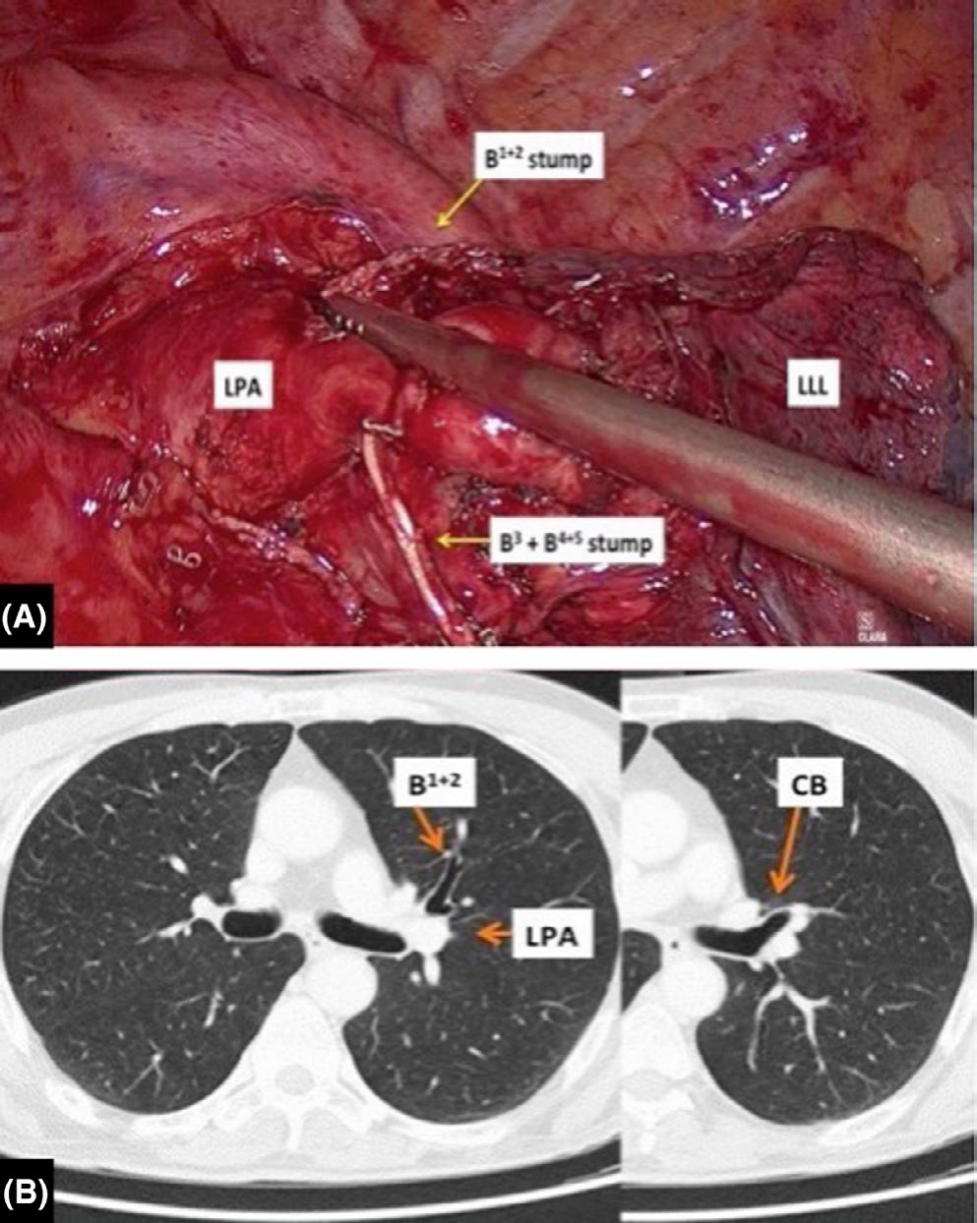
A, Intraoperative view after VATS left upper lobectomy. The left pulmonary artery passes between the stump of the apicoposterior segmental bronchus and the stump of the common bronchus to the anterior and lingular segments. B, Abnormal left pulmonary artery course identified on chest CT scans. The left pulmonary artery passes between a distinct apicoposterior segmental bronchus and the common bronchus to the anterior and lingular segments. B^1+2^, apicoposterior segmental bronchus; B^3^ + B^4+5^, anterior and lingular segmental bronchi; CB, common bronchus; LLL, left lower lobe; LPA, left pulmonary artery

A 55‐year‐old man underwent VATS left upper lobectomy for cT1b N0 M0 adenocarcinoma with a maximum standardized uptake value (SUV max) measured on fluorodeoxyglucose positron emission tomography (FDG‐PET) of 6,7. Under general anesthesia and double‐lumen intubation, a left VATS approach using three ports was performed. During the operation, we discovered a left pulmonary artery which passed between the apicoposterior segmental bronchus and the common bronchus to the anterior and lingular segments. Due to this vascular anomaly, it was necessary a separate closure of the segmental bronchi (Figure 1A). The anatomic variation was not recognized preoperatively but on review of the chest CT scan, it was clearly identified (Figure 1B). The patient had an uneventful postoperative course. The postoperative diagnosis was pT1b N0 M0 lung adenocarcinoma.

### 2.2 Case 2

**Figure 2:**
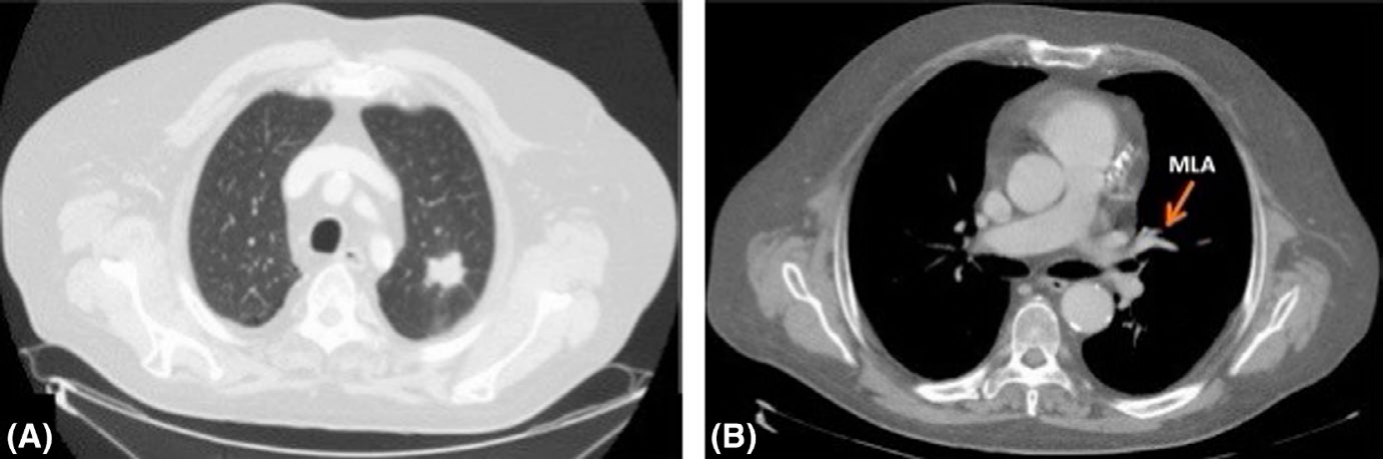
A, CT of the thorax with parenchymal window setting showing the left upper lobe lung cancer. B, Chest CT scan, viewed using mediastinal window, shows the so‐called mediastinal lingular artery arising from the anterior aspect of the mediastinal portion of the left pulmonary artery. MLA, mediastinal lingular artery

**Figure 3:**
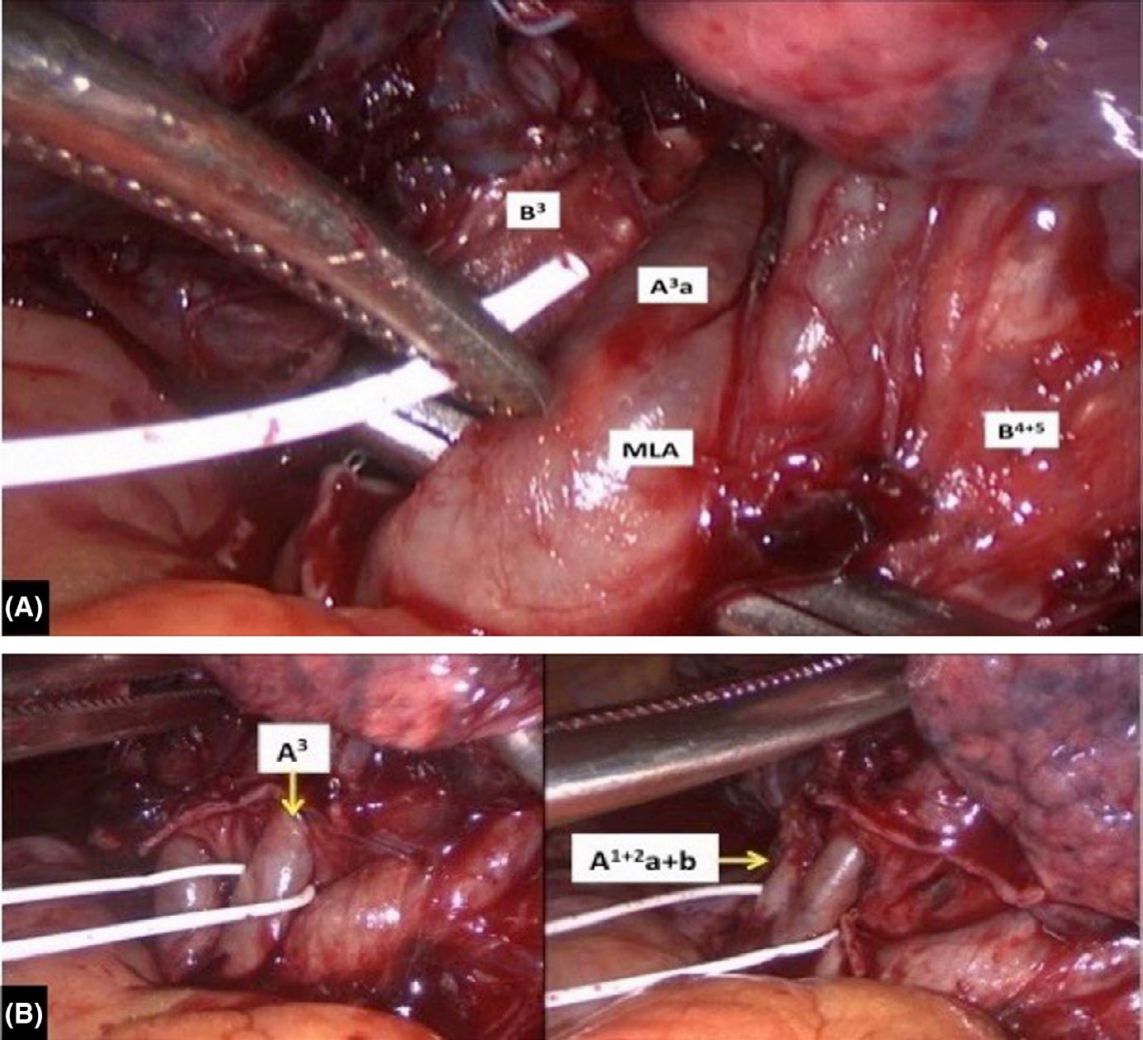
A, Isolation of a common trunk between the mediastinal lingular artery and the lateral branch of the anterior segmental artery which runs deeply into the lung tissue and at the back of the anterior segmental bronchus. B, Anterior segmental artery and branches of the apicoposterior segmental artery dissected and encircled with vessel loops. A^1+2^a+b, branches of the apicoposterior segmental artery; A^3^, anterior segmental artery; A^3^a, lateral branch of the anterior segmental artery; B^3^, anterior segmental bronchus; B^4+5^, lingular segmental bronchi; MLA, mediastinal lingular artery

A 71‐year‐old man was admitted to our department for treatment of adenocarcinoma located in the left upper lobe (Figure 2A). The clinical stage of cT1c N0 M0 indicated a surgical resection, and the patient was scheduled for a VATS left upper lobectomy. The procedure was done under general anesthesia with one lung ventilation and through three ports. Preoperative chest CT scans showed a lingular artery with mediastinal origin (Figure 2B) but during hilar dissection, we accidentally discovered a common trunk between the mediastinal lingular artery and the lateral branch of the anterior segmental artery, arising, with other upper lobe vessels, from the anterior aspect of the mediastinal portion of the left pulmonary artery (Figure 3A,B). The anomalous artery was divided using an endovascular stapling device, and the lobectomy was then completed by stapled division of all individual bronchovascular structures. The patient had an uneventful recovery and was discharged home on postoperative day 4. The final pathology showed a well‐differentiated adenocarcinoma without any involvement of hilar and mediastinal lymph node, measuring 28 mm in its maximal dimension.

## 3 DISCUSSION

The left main pulmonary artery usually curves around the back of the left upper lobe bronchus, continues as common basal trunk, and terminates in branches to the basal segments. In our patient, the left pulmonary artery passed between the apicoposterior segmental bronchus and the common bronchus to the anterior and lingular segments. This type of anomaly of the left pulmonary artery course, to the best of our knowledge, is not described in standard textbook of thoracic surgery. Only two similar case reports have been published in the recent international literature: In both cases, however, the authors discovered a left pulmonary artery which passed into the bifurcation between the lingular bronchus and the common bronchus to the left upper lobe anterior and apicoposterior segments.^5^, ^6^ This type of anomaly did not hamper proper dissection of hilar structures but simply required a separate closure of the segmental bronchi. This anatomic variation, unidentified preoperatively on chest CT scan, could lead to muddle during a left apico‐dorsal segmentectomy or a left upper lobe trisegmentectomy. The other case concerns a variation of the branching pattern of the left pulmonary artery. Recently, lingular artery branching patterns have been reported, with mediastinal origin in 9.2%, interlobar and mediastinal origin in 26.9%, and interlobar origin in 63.9%.^7^ In our patient, we identified intraoperatively a common trunk between the mediastinal lingular artery and the lateral branch of the anterior segmental artery. The knowledge of this vascular anomaly is important: When overlooked, it could lead to incorrect ligation of segmental arteries during a left anterior segmentectomy or lingulectomy. Keeping such anatomic variations in mind may help avoid inadvertent dissections and potential morbidity, especially during major lung resections performed in VATS due to less tactile inputs and tendency to only visualize the local surgical field.

## 4 CONCLUSION

Although few reports are available regarding variations of the course or variations of the branching patterns of the left pulmonary artery, a detailed knowledge of these anomalies is important for safer surgical procedures and a careful dissection is essential to avoid iatrogenic complications. As far as we know, the vascular anomalies described in this work and recognized in patients undergoing VATS left upper lobectomy have not previously been reported in the international literature.

## CONFLICT OF INTEREST

None declared.

## AUTHOR CONTRIBUTIONS

DA, UC, and DC: made contributions to the conception and design and performing manuscript preparation. CC, DA, and DC: performed the operation. PI, AS, MRV, and AI: performed data collection, data analysis, and interpretation. All authors reviewed and approved the final manuscript.
